# New Daily Persistent Headache in the Pediatric and Adolescent Population: An Updated Review

**DOI:** 10.3390/life14060724

**Published:** 2024-06-03

**Authors:** Paige Kalika, Teshamae S. Monteith

**Affiliations:** 1 Division of Pediatric Neurology, Department of Neurology, Miller School of Medicine, University of Miami, Miami, FL 33136, USA; 2 Division of Headache Medicine, Department of Neurology, Miller School of Medicine, University of Miami, Miami, FL 33136, USA; tmonteith@med.miami.edu

**Keywords:** new daily persistent headache, pediatric, adolescent, primary headache disorders, treatment

## Abstract

Purpose of review: New daily persistent headache (NDPH) is a primary headache disorder characterized by the sudden onset of continuous pain and its intractability to treatment. It is more prevalent in the pediatric population than the adult population, but remains understudied and underdiagnosed. The purpose of the current article is to provide a current overview of new daily persistent headache in the pediatric and adolescent population, including history, pathophysiology, clinical findings, current and emerging treatment options, and the results of recent studies and meta-analyses. Recent findings: Despite recent studies and meta-analyses showing significant phenotypic overlap between chronic migraine and NDPH in the pediatric population, multiple recent studies have come to conflicting conclusions about the overlap of medication overuse in headache and pediatric NDPH. Recent studies reveal alterations in neuroimaging, particularly in functional connectivity, in patients with NDPH. Patients frequently remain treatment-refractory even to medications that have historically proven helpful in this population; however, new treatment options, including calcitonin gene-related peptide (CGRP) monoclonal antibodies, may be more effective. Summary: NPDH remains a perplexing and difficult-to-manage condition for both children and adults. Despite a higher prevalence in the pediatric population, there are relatively few studies to guide the evaluation and treatment of NDPH in pediatric and adolescent patients. Early treatment, both pharmacological and non-pharmacological, should be employed to reduce disability. Overall, further studies are needed to better understand pathogenesis and to identify more effective therapeutic strategies, both pharmacological and non-pharmacological.

## 1. Introduction

New daily persistent headache (NDPH) is unique among headache syndromes as it is characterized by its onset—sudden, continuous, and unremitting—as opposed to its pathophysiology or other clinical characteristics. Patients remember, often to the date and hour, the moment that the headache began. Almost as characteristic as its onset is its intransigence; NDPH persists for at least 3 months and is notoriously resistant to treatment, which is a source of frustration for clinicians and patients alike. 

While NDPH was initially described in 1986 as a benign and often self-limited headache syndrome, later papers revealed its treatment-resistant nature. It was included in the ICHD-2 under “other primary headaches” [1], with the requirements that the headache becomes daily within 3 days of onset, is unremitting for more than 3 months, and has characteristics most resembling a tension-type headache (TTH) [2]. Despite this, a 2009 clinic-based pediatric study found that, although most attacks resembled TTH, an important proportion of patients with NDPH have frequent migraine symptoms [3].

In 2017, experts’ opinions on the ICHD-3 beta noted that NDPH is very common amongst adolescents but tends to be under-recognized and under evaluated, and also pointed out that the phenotypic similarities between NDPH and migraine should be noted in the ICHD-3 [4]. The overlap between NDPH and chronic migraine may be stronger in pediatric patients than in adults, but that does not confirm that pediatric NDPH and pediatric chronic migraine share a pathophysiology [5]. The aim of this review is to provide a recent update of NDPH in pediatric and adolescent populations.

## 2. Methods

**Figure 1:**
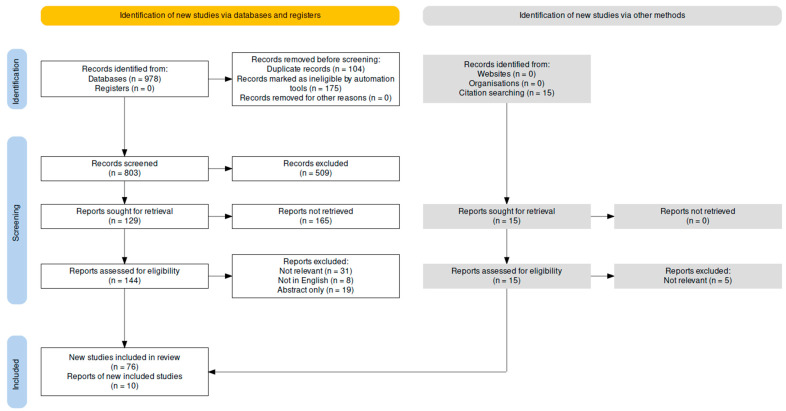
PRISMA flow diagram of study identification and inclusion.

We performed searches in PubMed, EMBASE, Scopus, and Web of Science for the keywords pediatric new daily persistent headache, adolescent new daily persistent headache, new daily persistent headache, and NDPH; 76 English language studies published between January 2009 and August 2023 were selected for review; and 2009 was chosen as a start date because it included the majority of studies focusing on or including pediatric NDPH while excluding older studies that were less relevant for an update in pediatric NDPH. A PRISMA flowchart of study identification is shown in Figure 1 [6]. Selected studies used in this review are shown in Table 1.

## 3. Results

### 3.1. Epidemiology

Chronic Daily Headache (CDH) affects 4–5% of adults and 1% of adolescents, with a prevalence of 2.4% in girls and 0.8% in boys [2] and an incidence of 1.13 per 100 person-years [17]. NDPH, a subtype of CDH, occurs in approximately 0.003% of the overall population [18]. Within the CDH population at tertiary centers, the prevalence of NDPH in children and adolescents ranges from 0.9 to 35%, compared to 1.7 to 10.8% in adults [19]. It maintains a female predominance throughout life, with a 2.5:1 female/male ratio in adults and a 1.8:1 female/male ratio in children and adolescents, and has an earlier age at onset in females (16–35) than in males (26–45) [2]. 

A 2021 study of pediatric patients at a headache program in a tertiary referral center diagnosed NDPH in 14% of the total headache population, 78% of whom were female and 22% male. For race/ethnicity, 73% of the NDPH patients were White, 18% identified as Black/African American, and 1% Asian. The median age was 14.8 years, and 76% of patients were between 12 and 18 years of age. Risk factors for NDPH in this study included White race, female sex, history of anxiety and/or depression, and pubertal age; in addition, 70% of patients reported a family history of headache disorders [20]. Almost 80% of children, compared with nearly 50% of adults, had an episodic headache disorder prior to the start of their continuous headache [7].

NDPH onset in pediatric and adolescent patients appears to have a seasonal pattern, with approximately 39% of all cases starting in September or January [21]. Also notable is that May showed a significant decrease in headache onset. This suggests that headache onset may be linked to returning to school after break and that the end of the school year is associated with a reduced risk of headache [21]. Interestingly, pediatric CDH is associated with school phobia, with no significant difference in terms of missed school days or medical or psychological factors seen between NDPH, transformed migraine, and comorbid chronic migraine (CM) and chronic tension-type headache (CTTH) [22]. 

### 3.2. Diagnostic Criteria and Clinical Findings

While some initial reports of NDPH included patients with migrainous features and the Silberstein–Lipton criteria focused on characteristics of headache onset rather than headache pain, ICHD-2 criteria for NDPH specifically excluded headaches with characteristics of migraine [23]. ICHD-3 criteria focus on onset and duration rather than specific headache characteristics, i.e., a persistent headache lasting for at least 3 months that occurs daily from its onset (Table 2). 

NDPH can have characteristics of migraine or tension-type headache or elements of both, and multiple studies of children and adolescents demonstrate significant overlap with features of CM. Papetti et al. found that 73% of children and adolescents with NDPH report migraine features, although nausea and vomiting are less common than in CM [12]. A 2021 study of patients presenting to a headache program in a pediatric tertiary care center found that the vast majority of patients reported experiencing migrainous features of some type, including photophobia (85%), phonophobia (85%), and a reduced activity level (88%) [20]. A 2023 adolescent study found that nearly all patients with NDPH reported headache characteristics consistent with migraine or probable migraine [5].

Patients can have a previous history of headache, but should not report worsening headache frequency before the start of the persistent headache and secondary causes must be ruled out. NDPH can overlap with medication-overuse headache (MOH), but only if the onset NDPH clearly predates the onset of medication overuse [24]. This is in contrast to CM, in which medication overuse (MO) is a common cause of transformation from the episodic subtype to the chronic subtype [25].

### 3.3. Precipitants and Triggers

Triggers are common in pediatric and adolescent NDPH. It was estimated that 88% of pediatric patients in a study reported a precipitating event, with the most common being a febrile illness (43%), preceding minor headache injury (23%), and cranial or extra-cranial surgery (10%) [8]. High altitude exposure has also been reported as a precipitant [20]. As noted, school stress is also closely linked to onset of NDPH, as cases peak in September and January, when students return to school [21].

Numerous infectious etiologies have been linked to pediatric NDPH, including Epstein–Barr virus, herpes simplex virus, cytomegalovirus, herpes zoster, adenovirus, toxoplasmosis, salmonella, strep, and *E. coli*, suggesting that a nonspecific inflammatory response to the preceding infection may be the trigger [8]. Recently, the SARS-CoV2 virus and sphenoiditis have also been reported as pediatric NDPH precipitants [26]. 

At least 40% of adult patients with NDPH report a trigger but, unlike in children, a seasonal pattern is not seen [7]. Similarly, emotionally stressful events, infections, and surgical treatment are commonly associated with NDPH in adults [8].

### 3.4. Pathogenesis, Secondary causes, and Mimics

The pathophysiology of NDPH remains unclear to date. An early study evaluating NDPH found an increased frequency of EBV excretion, suggesting that there may be an increased risk of EBV reactivation or susceptibility to EBV reactivation [27]. In another study of patients with NDPH, CM, and controls, cerebrospinal fluid levels of TNF alpha were elevated in individuals with NDPH and CM, but not for controls, suggesting the possibility that chronic inflammation exists in association with refractory headache [28]. It is also possible that, if the onset of NDPH symptoms is triggered by an inflammatory reaction, it could relate to the increased frequency of viral infections or a less developed immune system in children as compared to adults [7].

While phenotypic similarities with CM and CTTH are suggestive of a common etiology between them and NDPH, this does not necessarily implicate a similar pathophysiology or response to treatment. In support of common pathophysiology, a 2023 comparison of continuous headache in youth with migraine, NDPH, and persistent post-traumatic headache (PPTH) found that the majority of adolescent patients with PPTH and NDPH represent circumstances leading to the abrupt onset of migraine pathophysiology, suggesting that these patients had a predisposition to trigeminal nociceptive sensitization [5]. However, it is unclear if this is applicable to adults; as the peak incidence of migraine falls in adulthood, it may be that these triggers are more likely to uncover a previously unknown predisposition to migraine in children compared to adults [5]. 

A 2023 cluster analysis including pediatric and adolescent studies found that patients fell into three groups, one of which, based on age, sex, headache intensity, associated features, and triggerability, was very suggestive of migrainous biology, although the other two clusters were not [25]. This supports the concept that NDPH is a disorder with multiple etiologies, at least one of which could be linked to cortical hyperexcitability and sensitization of the trigeminovascular system.

Before diagnosing NDPH, it is important to rule out secondary and potentially treatable causes of persistent headache. Neuroimaging, evaluation of fundi, and, when appropriate, such as following a suspicion for raised intracranial pressure or infection, a lumbar puncture should be performed. Potential secondary causes are numerous and include post-traumatic headache, CNS infections, neoplasms, vascular events, congenital anomalies, vasculitis, idiopathic intracranial hypertension, spinal epidural venous congestion, and spontaneous CSF leak [29]. Secondary causes were identified in 23% of pediatric patients, all of whom had findings on neuroradiology or ophthalmological examination [8].

### 3.5. Co-Morbid Disorders and NDPH

As in other headache disorders, co-morbid symptoms are commonly seen in children with NDPH. In addition, nonspecific symptoms such as dizziness, generalized weakness, visual disturbances, abdominal pain, vomiting, sore neck muscles, and difficulty concentrating are common and bothersome [2].

Mental health screening is important in the approach for NDPH. Anxiety (19%) and depression (10%) are frequently seen, both before the onset of symptoms and after, as ongoing symptoms interfere with life and daily activities [2,20]. However, a 2023 cluster analysis including pediatric and adolescent studies showed that while both NDPH and transformed-CDH had high levels of headache-related disability, anxiety, and depression, diagnoses of anxiety and depression were slightly less common in NPDH, suggesting that NDPH does not have any more of a psychological factor than other forms of CDH [25]. This is in contrast to another study showing higher levels of somatization in NDPH; however, this study was specific to adult patients [30].

Sleep disturbance, including insomnia, and fatigue are very common, occurring in at least 2/3 of pediatric patients [2]. Interestingly, the relationship between sleep disturbance and disability was found to be greater in pediatric patients with NDPH and TTH than patients with CM, possibly because a cycle of daytime rest ineffective for symptom relief may lead to greater sleep disturbance at night and, in turn, to increased pain and fatigue the next day [31]. Further work is needed in this area, including in the role of continuous pain and circadian rhythm abnormalities. 

The relationship between medication overuse headache (MOH) and NDPH is not straightforward. This is because NDPH is a continuous headache, so assessing progression from baseline persistence can be challenging. Strong et al. report that 1/3 of the patients in their study fulfilled criteria for MOH and suggest that the poor response to abortive therapies predisposes these patients to MO, especially of non-steroidal anti-inflammatory drugs (NSAIDs). Papetti et al. report no evidence of a correlation between MOH and NDPH in pediatric patients, as withdrawal of the overused drugs did not improve the headache, which provides insight but is not diagnostic [8,20]. This is supported by Cheema et al., who also found that MO was much less common in NDPH than transformed CDH and speculated that this may be because the acute treatments are less effective [25]. Interestingly, MOH appears to be less common in children than in adults (19% vs. 41%) [7]. However, while Cheema et al. found that MOH was more common in NDPH than CM (22 vs. 33% in a childhood comparison study compared to 33 vs. 51% in an adult comparison study), Reidy et al. found no sub-group differences in age or MO in NDPH and CM [7,14], showing that this is a topic that needs further study. 

### 3.6. Imaging

While traditional neuroimaging in pediatric and adolescent NDPH has not been revealing in the past, recent studies have provided new insights. A 2022 study comparing pediatric NDPH patients to healthy controls found decreased cortical thickness and altered resting-state functional connectivity in areas involved in cognitive inhibitory control and pain-related processes [10]. Interestingly, a 2023 study used structural MRI to distinguish between adolescents with long COVID headache and those with primary headache disorders, including NDPH, revealing potential biomarkers for conditions that, so far, have only clinical criteria [32]. While a 2022 adult study did not show any gray matter differences in NDPH compared to healthy controls [33], a 2023 study investigating patients aged 14–70 showed changes in brain morphology such as cortical surface area, cortical thickness, and GM volume, accompanied by abnormal cortical neural activity [34]. A 2023 brain mapping study in adults showed abnormal functional connectivity in multiple brain regions involved in the perception and regulation of emotion and pain [35]. Interestingly, white matter abnormalities (WMA) and infarct-like lesions, which are often seen in migraine, have not been seen in NDPH [36]. Taken together, further work in advanced imaging is also needed in pediatric and adolescent age groups. 

### 3.7. Treatment

NDPH has been long recognized as resistant to treatment, although it may respond better when aggressive treatment is initiated early in the course of the disease [37]. As there is a paucity of targeted studies to help guide treatment in the pediatric age range, most clinicians initiate treatment based on the headache phenotype, typically when consistent with a presentation of migraine or tension-type characteristics.

#### 3.7.1. Acute Therapies

Acute treatment remains difficult (Table 3). A retrospective study showed that 35% of pediatric patients did not respond to symptomatic drugs like NSAIDs or triptans [8]. In addition, muscle relaxers such as tizanidine and anti-emetics such as ondansetron, prochlorperazine, and promethazine are commonly used. As APAP/butalbital/caffeine is also prescribed, education is needed to reduce the risk of MOH [38]. While occipital nerve blocks appear to be more effective in cluster headache and migraine, two studies show some efficacy in pediatric NDPH [16,39]. 

#### 3.7.2. Preventive Therapies

Given the persistent nature of the headache, prophylactic treatment is appropriate and should be initiated early in the course of the disease (Table 4). While there is little evidence for which medications are most effective in pediatric patients, a 2018 study of prescription patterns in pediatric headache, including 844 patients with a diagnosis of NDPH, found that amitriptyline and topiramate were the most frequently prescribed medications in pediatric NDPH, accounting for more than 10% of patients. Other medications, such as gabapentin, zonisamide, propranolol, verapamil, clonidine, nortriptyline, sertraline, citalopram, fluoxetine, escitalopram, venlafaxine, and cyproheptadine, were also commonly prescribed to pediatric patients with NDPH [38]. A 2022 retrospective study of 46 patients with NDPH showed that 80% showed at least a 50% reduction in headache days with preventive medication, but 20% showed no response [12].

OnabotulinumtoxinA has shown efficacy in the reduction of both the severity and frequency of headache in adults with NDPH, but there are no studies demonstrating efficacy in the pediatric population [19,42]. However, given the reduction in headache frequency and intensity seen in children with chronic migraine, along with its excellent safety profile [43], it is a reasonable option for patients who have not seen benefit from other therapies.

CGRP monoclonal antibodies targeting the ligand or its receptor are migraine-specific treatments that are established as effective for migraine prevention in both episodic and chronic migraine [44]. They are an exciting option that will hopefully be more readily available to pediatric and adolescent patients in the future. A chart review study of CGRP monoclonal antibodies in adolescents with refractory headache disorders, including NDPH, showed both subjective and functional improvement in patients with and without continuous headache at baseline [11]. Given that these patients had tried an average of 9.5 preventatives before the study, this is encouraging. Likewise, a retrospective chart review of eptinezumab in adolescents with chronic refractory headache, including two with NDPH, showed improvement in nine out of eleven patients [45]. 

Intravenous therapies should be considered given the level of refractoriness.

#### 3.7.3. Adjunctive Infusion Therapies

Repetitive IV sodium valproate did not change headache frequency in NDPH, but 1/5 patients had a decrease in baseline headache intensity from 6/10 to 5/10 and 2/5 patients had decreases in reported acute medication use [9]. Another study showed that DHE infusions resulted in at least some improvement for most patients with refractory headache regardless of headache type [13]. A case study showed a response to intravenous lidocaine in one adolescent who had not responded to previous treatments [19].

#### 3.7.4. Non-Pharmacological Interventions 

Given the medication-refractory nature of the condition, it is reasonable to add non-pharmacological approaches to the treatment regimen. Biobehavioral strategies like physical therapy, biofeedback, cognitive behavioral therapy, massage, acupuncture, and exercise can be helpful and are typically well tolerated. There has been a report of pain relief after osteopathic manipulative treatment [8] and another of improvement in NDPH frequency and intensity in two patients after the initiation of riboflavin [46]. Non-invasive neuromodulation is another favorable option due to its tolerable side effect profile, although studies are needed [19]. While there are no dietary or lifestyle guidelines developed specifically for patients with NDPH, given the significant overlap in clinical features and treatment options with chronic migraine, it is reasonable to extend similar recommendations focusing on stress management, sleep hygiene and behavioral interventions, good hydration, and regular healthy meals [19,47]. Likewise, as co-morbid depression and anxiety is commonly seen in patients with NDPH, mental health screening is needed so that appropriate multidisciplinary treatment can be initiated. 

### 3.8. Prognosis

Two subtypes of NDPH have been recognized: a self-limited form that resolves within several months but can relapse, and a treatment-refractory form that remains resistant even to aggressive treatment for over a year. Risk factors for long duration include migraine phenotype, a lack of a trigger, and a lack of prophylactic therapy [8]. The average duration of continuous pain was about 8 months, with 43% having a resolution of continuous pain within 6 months, 39% within 12 months, and 18% still having continuous pain after a year [8]. 

Between 16 and 66% of patients go into remission without treatment [48]. Studies in both pediatric and adult patients reflect both the self-limited and treatment-refractory subtypes, with some studies showing that NDPH does not interfere with activities and resolves spontaneously within 24 months, and others focusing on disability, treatment resistance, and poor prognosis [8].

A 2022 survey of pediatric headache clinicians found variability in how long patients with NDPH should be headache-free to be considered in remission [49]. This suggests that outcome measures in NDPH trials may need to be different from those commonly used in migraine trials, as headache days/month may not be the most appropriate primary outcome measure [49]. Other measures, such as headache intensity or headache-related disability, may prove more useful in demonstrating a meaningful improvement in quality of life.

## 4. Discussion

NDPH is a poorly understood primary headache disorder resulting in significant disability in pediatric and adolescent populations. Reidy et al. found no clinically meaningful difference in headache features and associated disability between adolescents with CM and adolescents with NDPH [14]. This differs from studies on adults, which show an overlap of migrainous features and NDPH in only 50 to 67% [20]. Even adults with NDPH who have features of CM have “less migrainous” headache than those with CM, as adult patients with CM tend to have an earlier age of onset, a family history of headache, and a higher number of associated migrainous symptoms [50]. This suggests that the etiology of adult and pediatric NDPH may differ and that there may be a percentage of NDPH cases in the pediatric and adolescent cohort that represents the sudden onset of a CM phenotype in a susceptible population.

Recent advances in neuroimaging suggest that changes in functional connectivity may provide the long-awaited biomarkers for primary headache disorders that have so far been absent in traditional structural neuroimaging. Further study is needed to turn these intriguing differences into research targets. Likewise, while evidence for effective treatments, both acute and preventative, remains scant, although CGRP monoclonal antibodies appear to have some efficacy and are beginning to be used more widely in the adolescent population. Given the typical intransigence of symptoms, early and aggressive treatment is recommended, and neuromodulation and other non-pharmacological options should be employed alongside traditional pharmacology as part of a comprehensive treatment plan. 

While the treatment of NDPH in the pediatric and adolescent population is, like many areas in pediatrics, guided mainly by adult-specific evidence and guidelines, a review of the current literature shows an encouraging uptick in publication. The three recent pediatric-inclusive [7,15,37] and one pediatric-specific [8] systemic and narrative reviews provide a comprehensive evaluation of the existing literature. 

Other single-center observational studies or retrospective reviews reviewed features and management of pediatric NDPH, either in isolation [12,20] or in comparison to CDH [5] or CM [14]. These studies have the advantage of relatively large sample sizes, especially for pediatric studies. Likewise, a retrospective chart review including 844 pediatric and adolescent patients with NDPH was helpful in revealing prescription patterns by diagnosis and suggesting avenues for further research, if not necessarily immediately helpful in directing treatment or revealing treatment efficacy [38].

While many of the other pediatric-focused studies looked at emerging treatment options, they were limited by sample size, as the studies involved relatively few patients and the numbers of NDPH patients within that sample was, with one exception [13], within the single to low-double digit range [9,11,45,46]. Likewise, while the studies showing potential biomarkers on MRI, functional connectivity, and brain mapping represent a fascinating and exciting new avenue in diagnosis, the sample sizes were still very low [32,34,35]. Further studies are needed to provide new targets for treatment and diagnosis, as well as outcome measures that may more accurately reflect meaningful changes in patient quality of life. Lastly, long term studies of NDPH are needed with pediatric onset to determine how headache may evolve into adulthood. 

## 5. Conclusions

NPDH is a disabling headache disorder impacting children, adolescents, and adults. There is significant overlap in phenotype with CM, but that does not necessarily confirm that there is shared pathophysiology; further studies and objective biomarkers are needed to elucidate any connections [5]. Advances in neuroimaging may provide these biomarkers, but further study is needed. Early treatment, both pharmacological and non-pharmacological, should be employed to reduce disability. Different patient-reported outcome measures may need to be considered in future studies. Overall, further studies are needed to better understand pathogenesis and to reveal more effective therapeutic strategies.

## Figures and Tables

**Table 1:** Selected studies of NDPH.

References	Type	Patient Samples or Surveys	Population Surveyed
Cheema S et al., 2023 [7]	Systematic review	2155 patients	Pediatric (13 studies) and adult (33 studies)
Patterson Gentile C et al., 2023 [5]	Cross-sectional study	150 (50 with NDPH)	Pediatric
Papetti, L et al., 2022 [8]	Systemic review	8 articles	Pediatric
Pavitt S et al., 2022 [9]	Retrospective review	45 (5 with NDPH)	Pediatric
Szabo, E et al., 2022 [10]	Pilot study	26	Pediatric
Greene KA et al., 2021 [11]	Chart review	112	Pediatric
Papetti L et al., 2021 [12]	Retrospective study	46	Pediatric
Srouji, R et al., 2021 [13]	Chart review	200 (51 with NDPH)	Pediatric
Reidy BL et al., 2020 [14]	Retrospective study	1170 (155 NDPH)	Pediatric
Yamani N et al., 2019 [15]	Systemic review	12 studies	Adult and pediatric
Puledda F et al., 2018 [16]	Chart review	159 (22 with NDPH)	Pediatric
Kung E et al., 2009 [3]	Chart review	187 (58 with NDPH)	Pediatric

**Table 2:** Diagnostic criteria adopted for the International Classification of Headache Disorders, 3rd edition [24].

**Description**
Persistent headache, daily from its onset, which is clearly remembered. The pain lacks characteristic features, and may be migraine-like or tension-type-like, or have elements of both.
**Diagnostic criteria**
A. Persistent headache fulfilling criteria B and C
B. Distinct and clearly remembered onset, with pain becoming continuous and unremitting within 24 h
C. Present for >3 months
D. Not better accounted for by another ICHD-3 diagnosis

ICHD-3: International Classification of Headache Disorders, 3rd edition.

**Table 3:** Options for acute treatment of pediatric NDPH.

Medication Class	Medications	Migraine-Specific Treatment	Non-Specific Treatment
NSAIDs	Ibuprofen	Yes ^1^	Yes ^3^
	Naproxen	Yes ^2^	Yes ^3^
Analgesic, non-opioid	Acetaminophen	Yes ^1^	Yes ^3^
Analgesic, non-opioid, barbiturate	APAP/butalbital/caffeine	No ^3^	Yes ^3^
Muscle relaxants	Tizanidine	No ^3^	Yes ^3^
Anti-emetics	Ondansetron	Yes ^3^	No
	Prochlorperazine	Yes ^3^	No
	Promethazine	Yes ^3^	No
Triptans	Rizatriptan	Yes ^1^	No
Triptan; NSAID	Sumatriptan/naproxen	Yes ^1^	No
Ergot derivative	Dihydroergotamine	Yes ^3^	No
Occipital nerve block	Lidocaine +/− dexamethasone	Yes ^3^	Yes ^3^

Abbreviations: NSAIDs, nonsteroidal anti-inflammatory drugs; APAP, acetaminophen. ^1^ Established efficacy in pediatrics [40,41]. ^2^ Probably effective in pediatrics [40,41]. ^3^ Evidence limited, use largely based on retrospective, observational studies or on studies on adults [40,41].

**Table 4:** Options for preventive treatment of pediatric NDPH.

Medication class	Medications	Evidence for Use in Migraine Phenotype	Evidence for Use in Tension-Type Phenotype
Anti-seizure medications	Topiramate	Yes ^2^	Yes ^3^
	Zonisamide	Yes ^3^	No
	Sodium valproate/valproic acid	Yes ^3^	No
	Gabapentin	Yes ^3^	Yes ^3^
	Lamotrigine	Yes ^3^	No
Anti-hypertensives	Propranolol	Yes ^2^	No
	Verapamil	Yes ^3^	No
Anti-depressants	Amitriptyline	Yes ^1^	Yes ^3^
	Venlafaxine	Yes ^3^	Yes ^3^
Antihistamine	Cyproheptadine	Yes ^3^	No
Neurotoxin	OnabotulinumtoxinA	Yes ^3^	No
CGRP monoclonal antibodies	Erenumab	Yes ^3^	No
	Fremanezumab	Yes ^3^	No
	Galcanezumab	Yes ^3^	No
	Eptinezumab	Yes ^3^	No

Abbreviations: CGRP, calcitonin gene-related peptide. ^1^ Established efficacy in pediatrics [41]. ^2^ Probably effective in pediatrics [41]. ^3^ Evidence limited, use largely based on retrospective, observational studies or in studies on adults [41].

## References

[1] Robbins M.S., Vanast W.J., Purdy R.A. (2017). New Daily Persistent Headache: Historical Review and an Interview with Dr. Walter Vanast. Headache.

[2] Baron E.P., Rothner A.D. (2010). New daily persistent headache in children and adolescents. Curr. Neurol. Neurosci. Rep..

[3] Kung E., Tepper S.J., Rapoport A.M., Sheftell F.D., Bigal M.E. (2009). New daily persistent headache in the paediatric population. Cephalalgia.

[4] Özge A., Faedda N., Abu-Arafeh I., Gelfand A.A., Goadsby P.J., Cuvellier J.C., Valeriani M., Sergeev A., Barlow K., Uludüz D. (2017). Experts’ opinion about the primary headache diagnostic criteria of the ICHD-3rd edition beta in children and adolescents. J. Headache Pain.

[5] Patterson Gentile C., Aguirre G.K., Hershey A.D., Szperka C.L. (2023). Comparison of continuous headache features in youth with migraine, new daily persistent headache, and persistent post-traumatic headache. Cephalalgia.

[6] Haddaway N.R., Page M.J., Pritchard C.C., McGuinness L.A. (2022). PRISMA2020: An R package and Shiny app for producing PRISMA 2020-compliant flow diagrams, with interactivity for optimised digital transparency and Open Synthesis. Campbell. Syst. Rev..

[7] Cheema S., Mehta D., Ray J.C., Hutton E.J., Matharu M.S. (2023). New daily persistent headache: A systematic review and meta-analysis. Cephalalgia.

[8] Papetti L., Sforza G., Frattale I., Tarantino S., Ursitti F., Ferilli M.A.N., Vigevano F., Valeriani M. (2022). The Enigma of New Daily Persistent Headache: What Solutions for Pediatric Age?. Curr. Pain. Headache Rep..

[9] Pavitt S., Gelfand A.A., Zorrilla N., Allen I., Riggins N. (2022). Efficacy and Safety of Repetitive Intravenous Sodium Valproate in Pediatric Patients With Refractory Chronic Headache Disorders: A Retrospective Review. Pediatr. Neurol..

[10] Szabo E., Chang Y.C., Shulman J., Sieberg C.B., Sethna N.F., Borsook D., Holmes S.A., Lebel A.A. (2022). Alterations in the structure and function of the brain in adolescents with new daily persistent headache: A pilot MRI study. Headache.

[11] Greene K.A., Gentile C.P., Szperka C.L., Yonker M., Gelfand A.A., Grimes B., Irwin S.L. (2021). Calcitonin Gene-Related Peptide Monoclonal Antibody Use for the Preventive Treatment of Refractory Headache Disorders in Adolescents. Pediatr. Neurol..

[12] Papetti L., Sforza G., Tarantino S., Moavero R., Ruscitto C., Ursitti F., Ferilli M.A.N., Vigevano F., Valeriani M. (2021). Features and Management of New Daily Persistent Headache in Developmental-Age Patients. Diagnostics.

[13] Srouji R., Schenkel S.R., Forbes P., Cahill J.E. (2021). Dihydroergotamine infusion for pediatric refractory headache: A retrospective chart review. Headache.

[14] Reidy B.L., Riddle E.J., Powers S.W., Slater S.K., Kacperski J., Kabbouche M.A., Hershey A.D. (2020). Clinic-based characterization of continuous headache in children and adolescents: Comparing youth with chronic migraine to those with new daily persistent headache. Cephalalgia.

[15] Yamani N., Olesen J. (2019). New daily persistent headache: A systematic review on an enigmatic disorder. J. Headache Pain.

[16] Puledda F., Goadsby P.J., Prabhakar P. (2018). Treatment of disabling headache with greater occipital nerve injections in a large population of childhood and adolescent patients: A service evaluation. J. Headache Pain..

[17] Connelly M., Sekhon S. (2019). Current perspectives on the development and treatment of chronic daily headache in children and adolescents. Pain. Manag..

[18] Grande R.B., Aaseth K., Lundqvist C., Russell M.B. (2009). Prevalence of new daily persistent headache in the general population. The Akershus study of chronic headache. Cephalalgia.

[19] Sadeghpour M., Abdolizadeh A., Yousefi P., Rastegar-Kashkouli A., Chitsaz A. (2023). New Daily Persistent Headache (NDPH): Unraveling the Complexities of Diagnosis, Pathophysiology, and Treatment. Curr. Pain. Headache Rep..

[20] Strong E., Pierce E.L., Langdon R., Strelzik J., McClintock W., Cameron M., Furda M., DiSabella M. (2021). New Daily Persistent Headache in a Pediatric Population. J. Child. Neurol..

[21] Grengs L.R., Mack K.J. (2016). New Daily Persistent Headache Is Most Likely to Begin at the Start of School. J. Child. Neurol..

[22] Fujita M., Fujiwara J., Maki T., Shibasaki K., Shigeta M., Nii J. (2009). Pediatric chronic daily headache associated with school phobia. Pediatr. Int..

[23] Bigal M.E., Lipton R.B. (2007). The differential diagnosis of chronic daily headaches: An algorithm-based approach. J. Headache Pain..

[24] (2018). Headache Classification Committee of the International Headache Society (IHS) The International Classification of Headache Disorders, 3rd edition. Cephalalgia.

[25] Cheema S., Stubberud A., Rantell K., Nachev P., Tronvik E., Matharu M. (2023). Phenotype of new daily persistent headache: Subtypes and comparison to transformed chronic daily headache. J. Headache Pain.

[26] Lee J., Rhee M., Suh E.S. (2015). New daily persistent headache with isolated sphenoiditis in children. Korean J. Pediatr..

[27] Diaz-Mitoma F., Vanast W.J., Tyrrell D.L. (1987). Increased frequency of Epstein-Barr virus excretion in patients with new daily persistent headaches. Lancet.

[28] Rozen T., Swidan S.Z. (2007). Elevation of CSF tumor necrosis factor alpha levels in new daily persistent headache and treatment refractory chronic migraine. Headache.

[29] Rozen T.D., Devcic Z., Lewis A.R., Sandhu S.J.S., Erben Y., Toskich B.B. (2023). A secondary daily persistent headache from onset with underlying nutcracker physiology and spinal epidural venous congestion: Case series with lumbar vein embolization as a therapeutic approach. Ther. Adv. Neurol. Disord..

[30] Uniyal R., Chhirolya R., Tripathi A., Mishra P., Paliwal V.K. (2022). Is new daily persistent headache a fallout of somatization? An observational study. Neurol. Sci..

[31] Rabner J., Kaczynski K.J., Simons L.E., LeBel A. (2018). Pediatric Headache and Sleep Disturbance: A Comparison of Diagnostic Groups. Headache.

[32] Kim M., Sim S., Yang J., Kim M. (2023). Multivariate prediction of long COVID headache in adolescents using gray matter structural MRI features. Front. Hum. Neurosci..

[33] Naegel S., Zeller J., Hougard A., Weise C.M., Hans Christoph D., Zuelow S., Kleinschnitz C., Obermann M., Solbach K., Holle D. (2022). No structural brain alterations in new daily persistent headache—A cross sectional VBM/SBM study. Cephalalgia.

[34] Qiu D., Wang W., Mei Y., Tang H., Yuan Z., Zhang P., Zhang Y., Yu X., Yang C., Wang Q. (2023). Brain structure and cortical activity changes of new daily persistent headache: Multimodal evidence from MEG/sMRI. J. Headache Pain.

[35] Wang W., Yuan Z., Zhang X., Bai X., Tang H., Mei Y., Qiu D., Zhang Y., Zhang P., Zhang X. (2023). Mapping the aberrant brain functional connectivity in new daily persistent headache: A resting-state functional magnetic resonance imaging study. J. Headache Pain.

[36] Rozen T.D. (2016). New daily persistent headache: A lack of an association with white matter abnormalities on neuroimaging. Cephalalgia.

[37] Peng K.P., Rozen T.D. (2023). Update in the understanding of new daily persistent headache. Cephalalgia.

[38] Rabner J., Ludwick A., LeBel A. (2018). Differences in Pediatric Headache Prescription Patterns by Diagnosis. Paediatr. Drugs.

[39] Gelfand A.A., Reider A.C., Goadsby P.J. (2014). Outcomes of greater occipital nerve injections in pediatric patients with chronic primary headache disorders. Pediatr. Neurol..

[40] Oskoui M., Pringsheim T., Billinghurst L., Potrebic S., Gersz E.M., Gloss D., Holler-Managan Y., Leininger E., Licking N., Mack K. (2019). Practice guideline update summary: Pharmacologic treatment for pediatric migraine prevention: Report of the Guideline Development, Dissemination, and Implementation Subcommittee of the American Academy of Neurology and the American Headache Society. Neurology.

[41] Szperka C. (2021). Headache in Children and Adolescents. Continuum.

[42] Ali A., Kriegler J., Tepper S., Vij B. (2019). New Daily Persistent Headache and OnabotulinumtoxinA Therapy. Clin. Neuropharmacol..

[43] Papetti L., Frattale I., Ursitti F., Sforza G., Monte G., Ferilli M.A.N., Tarantino S., Proietti Checchi M., Valeriani M. (2023). Real Life Data on OnabotulinumtoxinA for Treatment of Chronic Migraine in Pediatric Age. J. Clin. Med..

[44] Ailani J., Burch R.C., Robbins M.S., Board of Directors of the American Headache S. (2021). The American Headache Society Consensus Statement: Update on integrating new migraine treatments into clinical practice. Headache.

[45] Zorrilla N., Gelfand A.A., Irwin S.L. (2023). Eptinezumab for adolescents with chronic refractory headache: A retrospective chart review. Headache.

[46] Das R., Qubty W. (2021). Retrospective Observational Study on Riboflavin Prophylaxis in Child and Adolescent Migraine. Pediatr. Neurol..

[47] Nierenburg H., Newman L.C. (2016). Update on New Daily Persistent Headache. Curr. Treat. Options Neurol..

[48] Lobo R., Wang M., Lobo S., Bahra A. (2022). Time to retire ‘New daily persistent headache’: Mode of onset of chronic migraine and tension-type headache. Cephalalgia.

[49] Gelfand A.A., Szperka C.L. (2022). Diagnosing new daily persistent headache in children and adolescents: A survey of clinicians. Headache.

[50] Nagaraj K., Wei D.Y., Puledda F., Weng H.Y., Waheed S., Vandenbussche N., Ong J.J.Y., Goadsby P.J. (2022). Comparison and predictors of chronic migraine vs. new daily persistent headache presenting with a chronic migraine phenotype. Headache.

